# Non-human primate studies for cardiomyocyte transplantation—ready for translation?

**DOI:** 10.3389/fphar.2024.1408679

**Published:** 2024-06-17

**Authors:** Constantin von Bibra, Rabea Hinkel

**Affiliations:** ^1^ Institute for Animal Hygiene, Animal Welfare and Farm Animal Behavior, Stiftung Tieraerztliche Hochschule Hannover, University of Veterinary Medicine, Hanover, Germany; ^2^ Laboratory Animal Science Unit, German Primate Center, Leibniz Institute for Primate Research, Goettingen, Germany; ^3^ DZHK (German Centre of Cardiovascular Research), Partner Site Lower Saxony, Goettingen, Germany

**Keywords:** cardiac regeneration, cardiomyocyte transplantation, heart failure, myocardial infarction, large animal models, non-human primates, pluripotent stem cells

## Abstract

Non-human primates (NHP) are valuable models for late translational pre-clinical studies, often seen as a last step before clinical application. The unique similarity between NHPs and humans is often the subject of ethical concerns. However, it is precisely this analogy in anatomy, physiology, and the immune system that narrows the translational gap to other animal models in the cardiovascular field. Cell and gene therapy approaches are two dominant strategies investigated in the research field of cardiac regeneration. Focusing on the cell therapy approach, several xeno- and allogeneic cell transplantation studies with a translational motivation have been realized in macaque species. This is based on the pressing need for novel therapeutic options for heart failure patients. Stem cell-based remuscularization of the injured heart can be achieved via direct injection of cardiomyocytes (CMs) or patch application. Both CM delivery approaches are in the late preclinical stage, and the first clinical trials have started. However, are we already ready for the clinical area? The present review concentrates on CM transplantation studies conducted in NHPs, discusses the main sources and discoveries, and provides a perspective about human translation.

## 1 Introduction

Cardiovascular diseases are the primary cause of death worldwide, and the heart failure rate is still increasing (Khan et al., 2020; Bozkurt et al., 2023). Ischemic heart disease ranks as the most prevalent, which justifies the scientific desire to explore new treatment options for the injured heart. Current pharmacological treatments focus on the remaining myocardium to manage the symptoms by reducing the adverse remodeling process (Azevedo et al., 2016) but not reversing the process. The only curative treatment option for end-stage heart failure patients at the moment is heart transplantation. However, due to a limited donor pool and post-transplant complications, it is only an opportunity for a restricted patient population (Awad et al., 2022). Therefore, cardiac regenerative approaches have been studied intensively over the last decades (Eschenhagen et al., 2022; Garbern and Lee, 2022). Since induced pluripotent stem (iPS) cell-derived cardiomyocytes (CMs) are available in unlimited numbers (Takahashi and Yamanaka, 2006; Yu et al., 2007), one ambitious strategy became realistic: the transplantation of new CMs to the injured heart (Weinberger and Eschenhagen, 2021). Remuscularization of the damaged heart has been successfully approached in several small animal studies. Large grafts combined with beneficial functional outcomes were achieved with direct CM injection (Caspi et al., 2007; Laflamme et al., 2007; Shiba et al., 2012) and CM-containing patches (Zimmermann et al., 2006; Weinberger et al., 2016; Querdel et al., 2021). These promising results encouraged the field to move forward toward translation. Therefore, as a next step, large animal models are deemed indispensable for this therapeutic strategy prior to clinical translation (Dixon and Spinale, 2009; Chong and Murry, 2014). First, first-in-human clinical trials with healthy volunteers are not applicable for this kind of therapeutic approach. Second, the heart weight to cell number/patch size and the different routes of application cannot be addressed sufficiently in rodents. In addition, large animals better model human disease phenotypes due to their comparable anatomy and physiology (Plews et al., 2012; Hotham and Henson, 2020; Martínez-Falguera et al., 2021). The CM transplantation approach, therefore, has mainly been addressed in pigs and non-human primates (NHPs). The advantages of the pig model are the similar heart size and heart weight-to-body weight ratio, as well as the identical heart rate to humans (Lelovas et al., 2014; Romagnuolo et al., 2019). However, the establishment of a sufficient human transferable immunosuppression to avoid graft rejection seems challenging (Kawamura et al., 2012; Chong and Murry, 2014; Zimmermann, 2017). The other clinically highly relevant large animal model for CM transplantation is the non-human primate. Several groups translated the cardiac remuscularization approach to NHP models (key publications summarized in Table 1).

**TABLE 1:** Key publications of cardiomyocyte replacement therapy in non-human primates.

Study reference	NHP species	Age and weight (kg)	n-number and sex	MI induction method	Tx post MI (weeks)	Delivery approach	Cell source	Immunosuppression	Follow-up (weeks)
Gruh et al. (2024)	*M. fascicularis*	4–8 years, 7–13	n = 14 (15) (1f, 14 m)	Thoracotomy PL	2	Injection	hiCMA	MPred, ABC, and CsA	2, 12
Cheng et al. (2023)	*M. mulatta*	5–18 years, 9–11	n = 11 (m)	PCI or Thoracotomy I/R, 90 min	4	Injection	hiPSC-CM and EC	MPred, ABC, and Tac	4
Li et al. (2021)	*M. mulatta*	4–6 years, 7–14	n = 15 (17) (m)	Thoracotomy I/R, 180 min	0	Injection and i.v. and i.c.	hiPSC-CM	MPred, Tac, and MMF	4, 8, 12
Kashiyama et al. (2019)	*M. fascicularis*	6 years, 4–6	n = 12 (m)	Thoracotomy PL	2	Cardiac sheets	Allogeneic	Pred, Tac, and MMF	12, 24, 36
Liu et al. (2018)	*M. nemestrina*	6–15 years, 5–13	n = 9 (17), (1 m, 8f)	PCI I/R, 180 min	2	Injection	hESC-CM	MPred, CsA, and ABC	4, 12
Shiba et al. (2016)	*M. fascicularis*	4–5 years, 3	n = 10 (f)	Sternotomy I/R, 180 min	2	Injection	Allogeneic	MPred and Tac	12
Chong et al. (2014)	*M. nemestrina*	5–14 years, 9–12	n = 6 (7), (3 m, 3f)	PCI I/R, 90 min	2	Injection	hESC-CM	MPred, CsA, and ABC	2, 4, 12

Abbreviations: NHP, non-human primate; M, *Macaca*; kg, kilogram; m, male; f, female; MI, myocardial infarction; PCI, percutaneous coronary intervention; I/R, ischemia reperfusion injury; PL, permanent ligation; min, minutes; Tx, transplantation; i.v., intravenous; i.c., intracoronary, hiCMA, human-induced pluripotent stem cell-derived cardiomyocyte aggregates; hiPSC, human-induced pluripotent stem cells; hESC, human embryonic stem cells; CM, cardiomyocytes; EC, endothelial cells, MPred, methylprednisolone; Pred, prednisolone; CsA, cyclosporine A; ABC, abatacept; Tac, tacrolimus; MMF, mycophenolate mofetil.

This review will introduce the utilized NHP models, the applied myocardial infarction (MI) induction methods, the cell sources, and their delivery to the injured heart. Additionally, we will discuss the study designs of transplantation and follow-up timing. Finally, we will sum up the limitations and have a discussion on clinical obstacles and future deliberations.

## 2 Main

### 2.1 Non-human primate models

**FIGURE 1:**
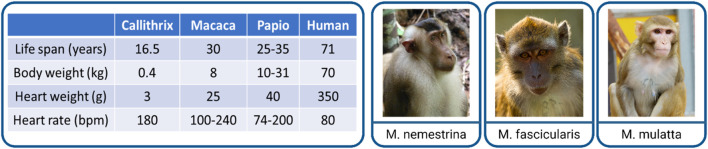
Non-human primates as a late preclinical model of cardiac remuscularization. Comparison of the main physiological parameters of NHPs to humans (left). Examples of utilized macaque species (right): *Macaca nemestrina* (pig-tailed macaque), *Macaca fascicularis* (cynomolgus macaque), and *Macaca mulatta* (rhesus macaque). Pictures taken (from left to right) by Carolin Kade, Chris Schloegl, and Margrit Hampe with the permission from the German Primate Center (DPZ).

Primates consist of more than 300 species, classified into three major categories: New World monkeys/*Platyrrhini*, Old World monkeys/*Catarrhini*, and others (T. Nakamura et al., 2021). Within the European Union, only non-human primates can be used for preclinical biomedical research due to their close phylogenetic background and similarities to human beings. NHP models still play an important role in translation and applied research, not only in the cardiovascular field. Within the broad variety of NHPs, the following species are the most utilized ones in biomedical research: common marmoset (*Callithrix jacchus*, New World monkey), cynomolgus macaque (*Macaca fascicularis*, Old World monkey), rhesus macaque (*Macaca mulatta*, Old World monkey), and baboons (*Papio* genus, e.g., *anubis* or *hamadryas*, Old World monkey) (Chatfield and Morton, 2018). The latter species are presently used primarily for solid organ or cardiac valve xenotransplantation studies. Their size/large scale is a key value to investigate pig heart to primate transplantation (Bailey, 2009; Längin et al., 2018). Marmosets are not suitable for transplantation purposes due to their small size (300–500 g, adult animals) and hematopoietic chimerism, which complicates the evaluation of the immune reaction (Silva et al., 2017). Non-human primates are conspicuously suitable for exploring MI-based treatment options due to their negligible collateral perfusion, similar to the human coronary network (Buss et al., 1983). Considering the size, phylogenetic similarities, and availability for translational, clinically relevant CM transplantation studies, macaques seem to be the best model to use. In addition, they are well-characterized, immunosuppression protocols are established, and a variety of assays and antibodies are available to analyze the heart. To investigate cardiac remuscularization, different macaque species were utilized (Figure 1). Which of the three macaque species was selected for use in the studies normally depends on the availability and experience of the individual institutes with the respective species.

In addition to the model organism (the NHP here), the choice of cell source for iPS cell-derived CMs is of importance for translational studies, as summarized in the question of allo- versus xenotransplantation, both with advantages and disadvantages. Allotransplantation would reflect the human clinical trial situation, because the cell source here is finally used, while using human cells means xenotransplantation, which might need intensified immunosuppression. However, for macaques, as well as for other NHP species, iPS cell-derived CMs are available and would allow for an allogeneic approach (Stauske et al., 2020; Rodriguez-Polo and Behr, 2022). Still, in most translational studies discussed in this review (Tables 1, 2), human iPS cell-derived CMs were used for transplantation; only in two of the studies was the allogeneic transplantation approach utilized (Shiba et al., 2016; Kashiyama et al., 2019). As shown in Table 1, homogeneity within these studies is less stringent as in rodent studies, which is reflected in a wide age range (4–18 years) and in the weight of the used animals. Heart failure is most prevalent among patients over 60 years of age (Bozkurt et al., 2023). However, CM transplantation studies in rodents mainly used young animals; therefore, adult animals (as used in the studies displayed in Table 1) might reflect more of the human situation, even though only animals over 15 years of age would be considered older. Nevertheless, the consequences of cardiac aging and CM senescence (Anderson et al., 2019; Salerno et al., 2022) have not been discussed in the displayed NHP studies. In addition to the age differences, variations in body weight (3–14kg, Table 1) should be emphasized. Body weight correlates with heart weight (Stahl, 1965); therefore, differences in heart size should be considered when discussing cell dosage. Variances of up to 8 kg in body weight within the studies could impact the calculation of sufficient cell quantities and thereby affect the effect size as well as the immune reaction and off-target effects. A general limitation in studies conducted in macaques is that almost exclusively male animals are used. The rational for this is that mainly male monkeys are available as young males have to be excluded from the breeding groups, while female animals are of utmost importance for the social structure of the breeding groups and therefore are rarely obtainable. It is, therefore, gratifying that female animals were included in more than one study. Although not specifically addressed in these studies, it is known from the field of cell transplantation that sex (mis)matches between the donor and recipient can affect the outcome (Kim et al., 2016; Ali et al., 2019). Therefore, when translating this to clinical trials, the sex of the donor versus recipient, in addition to the human leukocyte antigen (HLA) match, should be taken into account.

**TABLE 2:** Key publications of cardiomyocyte replacement therapy in non-human primates.

Study reference	Cell source	CM preparation	Transfer	Scar size	Input cell number	Graft size	Quantification engrafted CM
Gruh et al. (2024)	hiCMAs	Aggregates	10–12 i.m. injection	n/a	50mio	n/a	n/a
Cheng et al. (2023)	hiPSC-CM (and EC)	Single cells	4 i.m. injections	n/a	500mio CM (+500mio EC)	3%–5% of LV	n/a
Li et al. (2021)	hiPSC-CM	Single cells	10 i.m. injections	n/a	100mio/kg for i.m. application	n/a	n/a
Kashiyama et al. (2019)	Allogeneic	Cardiac sheets	4 epicardial sheets	n/a	4 × 3,6mio (14mio)	n/a	n/a
Liu et al. (2018)	hESC-CM	Single cells	15 i.m. injections	20% of LV	750mio	2% of LV 10% of Scar	22-126mio
Shiba et al. (2016)	Allogeneic	Single cells	10 i.m. injections	9% of LV	400mio	16% of Scar	n/a
Chong et al. (2014)	hESC-CM	Single cells	15 i.m. injections	5% of LV	1000mio	2% of LV	n/a

Abbreviations: hiCMA, human-induced pluripotent stem cell-derived cardiomyocyte aggregate; hiPSC, human-induced pluripotent stem cell; hESC, human embryonic stem cell; CM: cardiomyocyte; EC, endothelial cell; i.m, intramyocardial; LV, left ventricle; mio, million; kg, kilogram; n/a, not applicable.

### 2.2 Methods to induce myocardial infarction

**FIGURE 2:**
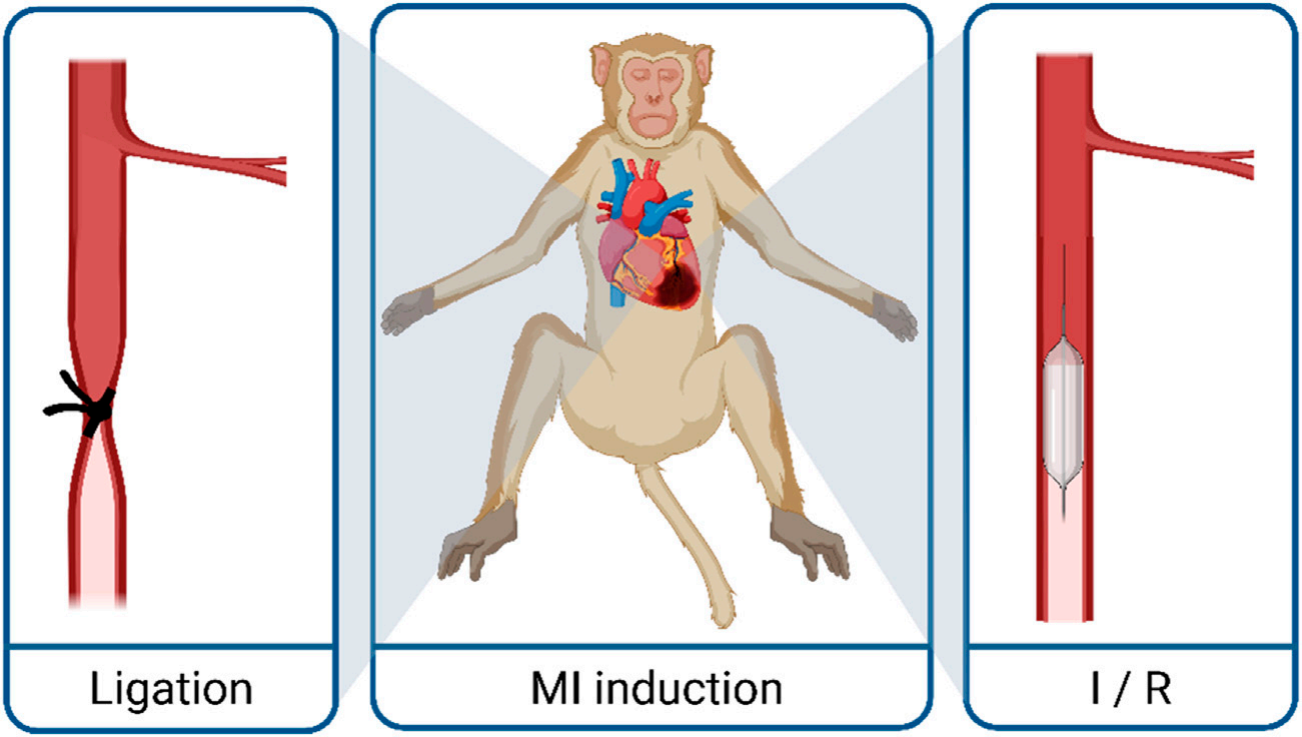
Techniques to generate myocardial infarction in macaques. MI, myocardial infarction; I/R, ischemia reperfusion.

Several procedures have been established to induce myocardial infarction (MI) in animal models (Martin et al., 2022). In comparison to ablation methods (e.g., cryoinjury), direct interventions on coronary arteries, such as the left anterior descending coronary artery (LAD), are thought to be the more clinically relevant model, reflecting MI in patients. In general, two models are used in this context: total occlusion of a coronary vessel and ischemia/reperfusion (I/R) injury, which is only a timely occlusion of the coronary artery. The later reflects more of the clinical situation since in patients, revascularization is the first choice of treatment. However, iPS cell-derived CM-transplantation seeks more treatment in the chronic phase (development of or reversal of heart failure) after myocardial infarction than the acute phase; therefore, we do not discuss the two different models and their impact on inflammation, scarring, and remodeling in the review. The different approaches of infarct induction in biomedical research are described as follows: induction of vessel occlusion can be achieved either surgically in an open-chest approach (via lateral thoracotomy or sternotomy) or via interventional catheter approaches (percutaneous transluminal coronary angioplasty, PTCA), both of which were used in the studies discussed in this review. The open-chest approach allows for either I/R or permanent occlusion via ligation, a procedure that has been studied in NHP for nearly a hundred years (de Waart et al., 1936). The advantage of a surgical approach is the direct visualization of the coronary artery to identify the correct position of the ligature and have a visually controlled target area of infarction after vessel occlusion (Shin et al., 2021). However, the surgical exposure of the heart is an invasive, painful procedure that includes a serious risk of infection. Tissue damage, especially the pericardial incision, leads to inflammation and epicardial fibrosis and thereby complicating re-operation for CM application in the surgical approach. Therefore, another access possibility could be considered: transluminal access is used to generate ischemic events through balloon inflation. The deflation of the balloon after a specific time (up to 180 min in NHPs, Table 1) results in a reperfusion. The catheter-based I/R is a minimally invasive strategy that circumvents the open chest. However, the identification of the desired occlusion location is more challenging, and anticoagulant and antiarrhythmic therapy is needed (Camacho et al., 2016). Furthermore, equipment needed for the catheter-based approach, an angiography system, is not available in all animal facilities, while the surgical approach does not require a specific equipment setup. From the perspective of animal welfare, the catheter-based, minimally invasive access seems to be advantageous since it is associated with less tissue damage and therefore less painful. The Murry group used the catheter-based approach, starting with an ischemic time of 90 min of the distal LAD in their first NHP study (Chong et al., 2014). However, the duration and position were insufficient to induce substantial damage, and only a minimal decline in global myocardial function was described (Liu et al., 2018). Therefore, in the second study, they chose a more extended period of ischemia (180 min) and occluded the coronary artery more proximal (mid-LAD) (Liu et al., 2018). The results demonstrated larger, transmural infarct scars with a clear decline in global myocardial function. Surprisingly, the increase in ischemic time did not lead to increased trop-out of animals in the second published study. The latest study by Cheng et al. generated functional impairment after 90 min occlusion time of the mid-LAD. Since interventional revascularization (percutaneous coronary intervention, PCI) has become a standard procedure for hospitalized patients with acute MI, the I/R model closely resembles their history. Nevertheless, there are still up to 30% of MI patients where no timely reperfusion is achievable (Cohen et al., 2010; Gharacholou et al., 2010), which is better reflected by the permanent ligation model. A further clinically relevant aspect is that the reperfusion itself causes additional damage (I/R injury) through a complex array of immune responses (Dorweiler et al., 2007; Yellon and Hausenloy, 2007). The differences in the subsequent inflammation and remodeling processes caused by these two different MI induction methods and the time point of transplantation after MI could influence the engraftment of the iPS cell-derived CMs; however, this has not been part of the investigations in NHPs so far. The induction techniques of permanent ligation and the I/R approach model the acute change from normal perfusion to complete vessel occlusion, mimicking therefore thrombotic, embolic or vasospastic etiologies. However, these causes are less common in humans than ischemia due to slowly progressing coronary atherosclerosis and stenosis, which can then be exacerbated by acute thrombotic occlusion or plaque rupture (DeWood et al., 1980; Burke and Virmani, 2007; Herrington et al., 2016; Severino et al., 2020). Both the permanent occlusion and the reperfusion approach (Figure 2) are clinically relevant.

However, since the used animals are rather young and healthy, no additional cardiovascular risk factors or co-morbidities were involved, and the occlusion occured suddenly. This only partially reflects the patient’s situation, and the results (especially functional improvement) need to be interpreted cautiously.

### 2.3 Cardiomyocyte delivery and engraftment

**FIGURE 3:**
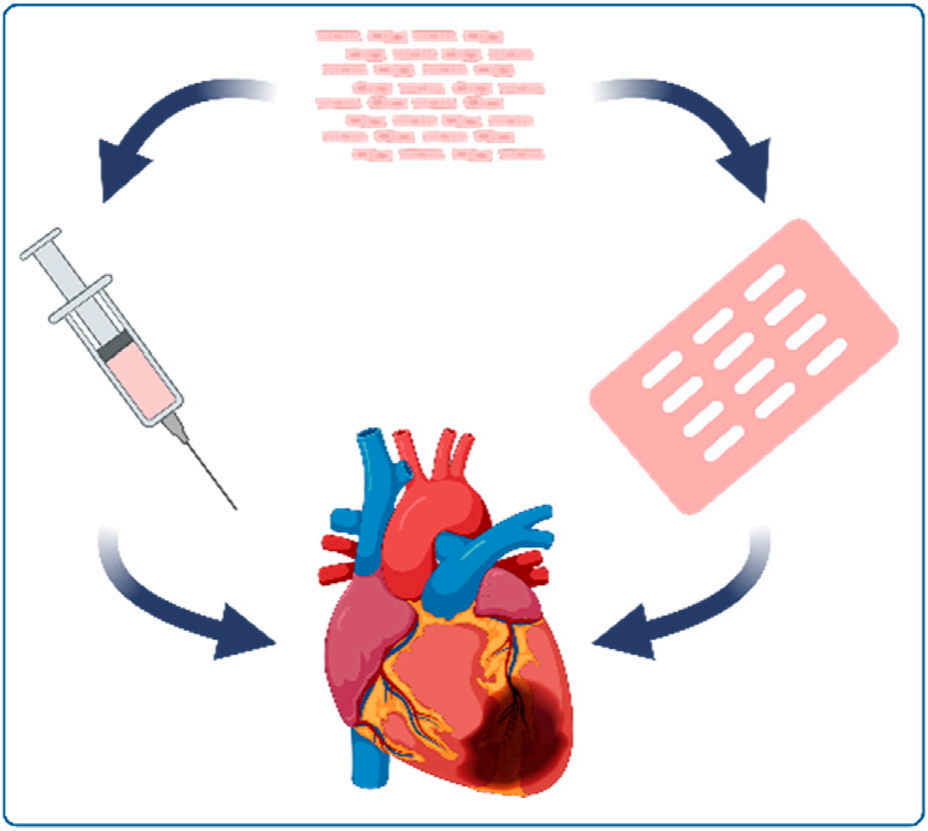
Main delivery approaches of cardiomyocyte (CM) transfer to the injured heart: intramyocardial CM injection and epicardial patch application.

In addition to the variation in ischemia duration and the kind of approach, the CM delivery is a second important technical aspect that needs to be taken into account. The major goal of the CM transplantation is to implant an effective number of cells at the site of interest with a good integration and survival of the transplanted cells. When reviewing methods to introduce new CMs to the heart, two main delivery routes have been accomplished: transplantation via intramyocardial (or intra-scar) injection (as single-cell solution or small aggregates) and the epicardial application of preformed 3D-constructs (Figure 3).

Both approaches carry their advantages and drawbacks (Feric and Radisic, 2016; Kadota and Shiba, 2019). Although efficacy was repeatedly proven in small animal models for both delivery approaches, the CM injection strategy has been mainly tested in NHP models (Tables 1, 2). This is thought to be more convenient for patient application because it can be performed minimally invasively via a catheter-based transluminal approach and does not necessarily require an open-chest approach. The study of Kashiyama et al. is the only publication that reported epicardial CM delivery via cell sheets in NHPs. The other remuscularization studies realized in NHPs focused on CM injection (Chong et al., 2014; Shiba et al., 2016; Liu et al., 2018; Cheng et al., 2023; Gruh et al., 2024). A recent project tested additional CM delivery approaches in cynomolgus monkeys (Li et al., 2021). Li et al. compared intracoronary, intravenous, and intramyocardial application of CMs and concluded that among the tested delivery strategies, intramyocardial injection is the most efficient delivery route for clinical purposes.

The CM transplantation studies followed the hypothesis that functional recovery is based on repopulated force-generating CMs. This implies that a substantial remuscularization must be achieved to validate their therapeutic potential. As mentioned previously, sufficient immunosuppression is required to afford cell engraftment in the xeno- and allogeneic approach. Based on clinical organ transplant treatment, different combinations of immunosuppression drugs (Table 1) have been successfully applied in terms of CM survival. Graft size also depends on the input cell number (Querdel et al., 2021). The tested CM numbers per macaque heart ranged from 50 million to one billion cells via 4 to 15 injections per heart (Table 2).

Kashiyama et al. applied 14 million cells spread over four cardiac sheets. These publications reported the graft-related repopulation of CMs. Evaluation of the graft size was only performed in some macaque studies (Chong et al., 2014; Shiba et al., 2016; Liu et al., 2018) and exhibited that CM injection resulted in a remuscularization of up to 5% of the left ventricle, which is comparable with the achievements in small animal studies (Eschenhagen et al., 2022). However, most studies lack a quantification of cell mass and number at the end of the experiments (Table 2) to better investigate cell survival and the engraftment of different approaches. In further studies, the focus should be not only on safety and functional improvement but also on cell survival, gain of myocardial mass, and coupling/engraftment of the cells in the host myocardium.

### 2.4 Cell sources

In addition to the delivery method, the cell source is an important aspect in terms of an adequate engraftment and potential side effects of different cell types. The cell-based field of cardiac regeneration tested already different cell sources. In addition to differentiated CMs, iPS cells have also been applied for transplantation in small animals. Beneficial effects were observed when iPS cells were delivered via injection and with a patch approach (Nelson et al., 2009; Dai et al., 2011). To reduce the teratogenic risk of iPS cells, cardiovascular progenitor cells (CVPCs) were also part of the investigations (Vicinanza et al., 2017; Wang et al., 2017; Monsanto et al., 2020). However, the only study which addressed CVPC in NHPs reported a lack of remuscularization (Zhu et al., 2018). Herein, we focus on the cell type of this review: the widely used stem cell-derived cardiomyocytes. The first CM transplantation study described in NHPs used human embryonic stem cell (hESC)-derived CMs (Chong et al., 2014). In order to overcome legal, political, and ethical concerns associated with human embryos, the field has evolved in the direction of human iPS cells as a basis for CM generation (Aboul-Soud et al., 2021). The best exogenously cell source to avoid immunological cell rejection would be individual patient-derived (=autologous) CMs. Currently, the autologous approach does not seem applicable for a broad clinical use. High costs, the time-consuming process of cell-line generation, and regulatory hurdles limit it since an individual cell line would be counted as ATMP (advanced therapy medicinal product) and therefore has to fulfill all safety and functionality requirements. From a clinical perspective, it is comprehensible that CMs of human origin were generated and tested for transplantation purposes. Most preclinical studies are based on the xenogenic background, where human cells were transplanted. Nevertheless, this might require more intense immunosuppression and does not reflect the clinical situation. However, as an equivalent to the clinical phase I trial, an allogeneic approach was used in two studies, where CMs derived from macaque iPS cells were used. Shiba and coworkers demonstrated that allogeneic major histocompatibility complex (MHC)-matched CM transplantation is feasible with immunosuppression, and the engrafted CM survived the observation period (12 weeks). The second study, which addressed the allogeneic approach via cardiac sheet application, described cell survival for both MHC-matched and mismatched recipients. Unfortunately, no more transplanted CMs were detectable in the group with the most extended follow-up period of 6 months (Kashiyama et al., 2019). Regardless of the cell origin (xeno- or allogeneic), the studies displayed that immunosuppression still seems unavoidable.

### 2.5 Study designs: timing of transplantation and follow-up

Previous studies in small animals mainly transplanted CMs in the early stage after injury and were analyzed after a short follow-up period (Zimmermann et al., 2006; Laflamme et al., 2007; Caspi et al., 2007; Shiba et al., 2012; Funakoshi et al., 2016; Weinberger et al., 2016; W. Zhu et al., 2018; Munarin et al., 2020; Sun et al., 2020; Jabbour et al., 2021; Querdel et al., 2021). In this setting, promising results were obtained: partial scar remuscularization resulted in functional improvement. For a more basic research approach, these proof-of-principal studies with early cell transplantation were sufficient to address the fundamental questions of cell survival and functional benefit after (sub-)acute myocardial infarction. However, since the widespread access to reperfusion therapy, more and more patients survive an acute MI event and develop heart failure over time (Heusch et al., 2014; Heusch and Gersh, 2017). The transplantation early after MI does not resemble well the most likely clinical application for these regenerative approaches (Eschenhagen et al., 2022). To narrow the gap to the clinical scenario of patients with advanced heart failure, transplantation was performed in the chronic stage after injury in selected rodent studies (Fernandes et al., 2010; Shiba et al., 2014; Riegler et al., 2015; von Bibra et al., 2022). Irrespective of the CM delivery route (injection and patch application), the outcomes were almost identical: transplantation is less efficient than that in the subacute injury models. Grafts were smaller, and no significant increase in functional parameters was observed. In the proof-of-concept studies performed in NHPs, where the goal should be to represent the potential human application as effectively as possible, it was surprising that, apart from one study (Cheng et al., 4 weeks), only the early injury (0 days and 2 weeks) was again the subject of investigation. This does not reflect the anticipated CM therapy as a last resort for patients with chronic ischemic heart failure (Kadota et al., 2020; Silver et al., 2021). To phrase it more provocatively, it is understandable that the subacute transplantation setting was used again because better results can be expected here. However, the contribution to narrowing the translational gap is debatable in this acute to subacute phase after MI. Inflammation, remodeling, and the development of heart failure are still ongoing and might influence engraftment, cell survival, and partly masks the beneficial effects of the transplanted CMs. In regard to investigating long-term survival of the transplanted CMs, some NHP studies included extended follow-up periods (Table 1). Animals treated with injected CMs were observed for up to 3 months, and cells survived with an efficient immunosuppression regimen. The transplantation of cardiac sheets was monitored for up to half a year, with the less encouraging observation of chronic rejection.

## 3 NHP study achievements and translational impediments

The preclinical investigations of cardiac remuscularization therapy advanced considerably since NHP models entered the validation process. Since 2014, several publications evaluated the transplantation of *in vitro*-generated CM in injured macaque hearts. The key benefit of these studies is undoubtedly the translational value due to the proximity to humans. Central achievements were gained in the clinically predictive, human-like NHP model. The *in vitro* generation of iPSC-derived CMs is at an advanced technological level (Lyra-Leite et al., 2022). Application in NHPs demonstrated that clinically scalable amounts of CMs with a high purity can be produced and applied to the injured heart. Induction of myocardial infarction was created via permanent or transient LAD occlusion, mimicking the human’s MI scenario and simulating closely clinical reality. Substantial damage of the heart with functional impairment was generated and therefore opened a therapeutic window. Sufficient immunosuppression regimen, in clinically relevant doses, enabled xeno- and allogeneic transplanted cells to survive over months. Safety was demonstrated over the post-transplant observation period, and no teratoma or abnormal cell growth has been reported.

To summarize it with a more global picture, CM transplantation resulted in a substantial remuscularization of the injured macaque hearts, and an amelioration of function was repeatedly demonstrated. These findings can be considered encouraging for the translational field and led to clinical translation. Now, more than five clinical trials using iPS cell-derived CMs in heart failure patients are ongoing (*ClinicalTrials.gov*).

However, some limitations should be discussed regarding the macaque model.

Limited availability of these animals and ethical and economic conflicts led to studies with small sample sizes (Table 1). This implicates a high standard deviation, with a limited statistical outcome in evaluating efficacy. Both induction methods of MI in the NHP models generated an acute-to-normal rather than an acute-to-chronic vessel occlusion, therefore lacking the ability to mimic the history of atherosclerosis and endothelial dysfunction that is frequently displayed in patients.

NHPs are often regarded as the ideal model of translation. In comparison to other large animal models (e.g., pigs), the macaque species are smaller in size. According to that, the heart has 1/10 of the weight of a human adult heart (Gandolfi et al., 2011; Chong and Murry, 2014). In particular, regarding dose-finding studies, the macaques allow only limited investigations. Calculations to determine an effective cell amount for human application should be carried out carefully (Eschenhagen et al., 2022).

To return to the original question: Is the CM transplantation approach ready for clinical application? No, because aside from the translational achievements ascertained in NHP models, relevant clinical impediments need to be discussed, addressed, and resolved first:

Since large animal models have been implemented in the preclinical investigation of cardiac remuscularization, engraftment arrhythmias (EA) were frequently reported (Chong et al., 2014; Shiba et al., 2016; Liu et al., 2018). These observations of post-transplant arrhythmias emphasise the importance of large animal models for preclinical validation, as these studies have additional value to the rodent results. EAs are discussed at the moment as one of the most concerning barrier toward translation (Eschenhagen et al., 2017). This ventricular tachycardia occurred transiently, mainly in the first weeks after CM transplantation. Hence, the immaturity of the implanted CMs seems reasonable to cause this focal automaticity. To suppress these potentially life-threatening EAs, pharmacological treatment has already been investigated in pigs (K. Nakamura et al., 2021). In addition, approaches to enhance CM maturation prior to *in vivo* application could tackle the issue (Karbassi and Murry, 2022).

The consensus in the research community that the allogeneic approach will be most likely applicable in the clinic harbors the immunological dilemma. Even if MHC-matched donors were selected for allogeneic transplantation, therapy with immunosuppressants is still necessary to avoid graft rejection (Shiba et al., 2016; Kashiyama et al., 2019). Further investigations in evaluating concentrations and ideal combinations are important due to the fact that the long-term treatment of gravely ill heart failure patients can result in severe side effects, and the immunosuppression itself could have an impact on the MI disease pathway (Ruiz and Kirk, 2015; Diehl et al., 2016; Demkes et al., 2021). A more elegant way of avoiding immune rejection has emerged with the generation of hypoimmunogenic cell lines (Deuse et al., 2019). The application of gene-edited CMs that can evade the immune system is a highly desirable alternative to immunocompromising agents (Lanza et al., 2019; Sung et al., 2023).

An additional issue is still the poor cell survival and the low engraftment rate (Robey et al., 2008). One approach to improve cell retention after injection has recently been demonstrated in cynomolgus monkeys. The co-transplantation of endothelial cells substantially enlarged graft size (Cheng et al., 2023). Cell survival can also be limited by the application route. Macaque studies mainly addressed the CM injection, where a major discussed drawback is the direct cell washout after injection (Chong and Murry, 2014; Martens et al., 2014). Patch approaches are often discussed as the delivery alternative to prevent a high cell loss after injection (Huang et al., 2020; Li et al., 2021; Yu et al., 2023). A cell survival comparison with patch-based CM application is, due to the sparse publication record, not possible in NHPs so far. To overcome the limitations of each delivery approach and to synergize their benefits, one stimulating idea would be to combine both strategies.

The so-called intramyocardial injection of CMs is frequently displayed in NHP studies. Nevertheless, the global idea is to remuscularize the damaged part (scar) of the heart and not to create hyperplasia in the viable myocardium. For most of the studies, the intramyocardial injection is indeed the correct term because the presented grafts are frequently surrounded by the vital myocardium. The neologistic term of intrascar injection would better reflect the aim of the approach, which is remuscularization, not hypermuscularization. Unfortunately, inadequate application to the infarct area was not only a limitation of the tiny hearts of small mammals, it was also evident or apparent in large animal NHP studies. In future projects, technical approaches that result in injecting the CMs primarily in the scar (and of course in the border zone) rather than generating additional myocardium in the viable zone should be addressed.

As mentioned previously, most of the NHP studies transplanted CMs in an early (sub-)acute stage of injury. Therefore, the translational question, of how successful will the engraftment be when targeting chronically injured hearts in patients, is still open. For future perspective, one idea to improve the transplantation success in the chronic setting could be to identify beneficial pathways present in the subacute injury. Identified targets could be included in the patch or injection medium or eventually used to pretreat the injured heart prior to transplantation. The chronically injured heart is more hostile for CM transplantation because of the absence of inflammatory cells and the stiff collagenous scar with a low vascular density (von Bibra et al., 2022). In other words, the subacute setting could be more likely because of the ongoing remodeling process with inflammation and neoangiogenesis. Targets of these pathways could be used to improve the transplantation success in the clinically relevant chronic setting. For a clinical application, the long-term graft maintenance is essential. The only study that included a follow-up observation of 6 months reported chronic rejection. Longer follow-up studies are also needed to scrutinize the risk of tumor growth.

In spite of the advanced translational progress gained with the NHP studies, numerous demands remain and need further investigation. However, cardiac remuscularization is currently at an exciting stage; the intensive preclinical work has already led to the first clinical trials. According to ClinicalTrials.gov, more than five clinical trials are running at the moment to evaluate the therapeutic potential of CM transplantation. Even though the studies conducted in NHPs mainly investigated the CM injection, a variety of delivery strategies are approached in the clinical trials. The HEAL-CHF trial (NTC03763136) from China is set up to test the intramyocardial injection of CMs during coronary artery bypass grafting. In addition to the epicardial injection, the same group is testing an alternative access for intramyocardial injection. Via a catheter-based endocardial application, different CM doses are injected (NTC04982081). The German BioVAT-HF trial (NCT04396899) used engineered heart muscles as an epicardial patch approach. The collagen-based tissue contains, in addition to iPS cell-derived CMs, stromal cells. The Japanese LAPiS trial (NTC04645018) evaluated the safety of CM spheroid. In addition, a case report from Japan (#jRCT205319008) described recently the successful transplantation of CM-containing patches (Miyagawa et al., 2022). Fortunately, no adverse events (e.g., arrhythmias and tumor growth) were detected. However, immunosuppression was suspended 3 months after transplantation.

 In summary, first steps toward clinical application are done in iPS cell-derived CM transplantation. However, these are all early clinical trials, with low patient numbers, and only a few centers participating in these trials. Therefore, larger clinical trials have to be performed before bringing this approach to a broad clinical application. The field, however, is, in our view, moving in the right direction, and late translation seems to be possible.
